# Gender predisposition in patient attitudes and preferences during dental treatment – A mixed method study from Lahore, Pakistan

**DOI:** 10.12669/pjms.41.10.11952

**Published:** 2025-10

**Authors:** Afifa Ehsan, Eesha Aftab, Muhammad Ahmad Jawwad Khan, Narmeen Azhar, Iqra Ayub, Ali Sajjad

**Affiliations:** 1 Prof. Dr. Afifa Ehsan, BDS, MPhil, CMT, CME, CMJE, DHEP, MHPE, Professor, Department of Oral Biology, Faryal Dental College, Lahore, Pakistan; 2 Eesha Aftab, 4^th^ Year BDS Student, Fatima Memorial College of Dentistry, Lahore, Pakistan; 3 Muhammad Ahmad Jawwad Khan, 4^th^ Year BDS Student, Fatima Memorial College of Dentistry, Lahore, Pakistan; 4 Narmeen Azhar, 4^th^ Year BDS Student, Fatima Memorial College of Dentistry, Lahore, Pakistan; 5 Iqra Ayub, 4^th^ Year BDS Student, Fatima Memorial College of Dentistry, Lahore, Pakistan; 6 Ali Sajjad, 4^th^ Year BDS Student, Fatima Memorial College of Dentistry, Lahore, Pakistan

**Keywords:** Dentist gender, Doctor-patient relationship, Gender bias, Pakistan, Patient preferences, Perception, Qualitative study, Stereotyping, Tertiary care

## Abstract

**Objective:**

To investigate predispositions, focusing on the impact of the gender of dentists on patient attitudes and exploring how these predispositions affect dental care.

**Methods:**

A cross-sectional, mixed-method study was planned that surveyed 383 patients from July 2023 to July 2024 at three private dental hospitals in Lahore, Pakistan. A combination of quantitative and qualitative data collection techniques was used, including thematically transcribing recordings to identify recurring themes related to gender predispositions in dental care.

**Results:**

The study found significant gender-based preferences; male dentists were preferred for attributes like decisiveness, experience, and time management, with 37% (n=142) respondents viewing them as more confident and experienced. Female dentists were favored for aesthetics, sensitivity, and preventive care, with 44% (n=172) appreciating their attention to detail and 52% (n=201) valuing their sensitivity and caring nature. Substantial associations were seen for attention to aesthetics (p ≤ 0.04), concern for cleanliness (p ≤ 0.03), multiple visits (p ≤ 0.00), sharing dental fears (p ≤ 0.00), discussing general health (p ≤ 0.00) and financial issues (p ≤ 0.00), and understanding problems (p ≤ 0.00). Qualitative analysis found preferences under five themes including comfort level, perception of competence, cultural and religious factors, trust and confidence in treatment, bias and stereotypes.

**Conclusion:**

The study findings highlight gender predispositions in dental care, influenced by cultural stereotypes. Addressing these biases is crucial for improving patient satisfaction and ensuring equitable treatment. The study further suggests that both patients and dental professionals should focus on qualifications and skills rather than gender, with further research needed to explore the deeper impact of these predispositions on patient perceptions.

## INTRODUCTION

All human beings are biologically born as male or female however; as they mature, they internalize and exhibit behaviors, attitudes, roles, activities, expectations, and desires influenced by societal norms and expectations.^1^ These attributes of societal norms assign the roles and behaviors of girls or boys and men or women. These roles termed as gender are a major determinant today for configurations of human endeavor, including health care.^2^ In today’s modern world, where technology is taking over the world with daily innovation, people live in very divisive times. While social media may create the illusion that society has made significant progress in addressing issues like sexism, racism, and gender inequality, many people still hesitate to express their views out of fear of conflict or professional repercussions.^3^

Along with all these advancements, the gap between the patient and the dentist persists. History clearly states that there was a time when doctors decided the treatment for the patient. Living in the twenty-first century this trend has shifted more towards a patient-centered approach where the patient decides his treatment majorly (Carl Roger’s concept of psychotherapeutics).^3^ Earlier dentistry was considered a male-dominated field however; in the last few decades, a significant number of women have entered this profession.^4^ Each dental professional acquires different attributes depending on where they were born, their cultural standing and their social status quo.^5^ Women commonly have more kindness and empathy-related traits.^6^ whereas men are considered to be more confident, intimidating, and skilled than females.^7^

Nevertheless; healthcare professionals have a duty to treat every patient equally. Correspondingly, patients should also have equal preference for trained, competent, and skilled male or female dentists despite any gender predispositions.^2^ Subsequently, this study was designed to evaluate these predispositions in a country with a population of 253 million and a total of 31,867 registered dental practitioners in Pakistan out of which 33.5% are males and 66.5% are female dental practitioners, observed in a study carried in 2022.^8^,^9^ Consequently, the objective of the present study was to analyze the reasons for the incorporation of gender competencies and predispositions in dental hospitals and to review ways to in-build gender competencies and their assessment.

## METHODS

This cross-sectional study utilized a mixed-method approach with an explanatory sequential design, combining both quantitative and qualitative data collection techniques to investigate gender predispositions in patient attitudes and preferences regarding dental treatment.^10^ The study design followed GRAMMS (Good Reporting of a Mixed Methods Study) guidelines. The study sample was collected by convenience sampling and the study duration was from July 2023 to July 2024 at three private dental hospitals in Lahore, Pakistan. The study targeted adult patients aged between 18 and 70 years who had undergone dental treatment. Participants were excluded from the data collection process who had not undergone any dental treatment, had negligible experience with a dental practitioner, and were unwilling to participate. Informed consent was obtained from all participants before the survey and participants were assured of confidentiality and anonymity, with data stored securely and used solely for research purposes.

### Ethical approval:

The study was approved by the Research Committee of FMH College of Medicine & Dentistry, Lahore, Pakistan (IRB # FMH-25/05/2023-IRB-1218, dated: July 3, 2023).

### Data collection:

***1. Survey instrument:*** Based on a thorough literature review, a questionnaire comprising both closed and open-ended questions to collect quantitative data and qualitative insights was developed. Content validation of the questionnaire was done by experts and a pilot study was conducted before the start of the research. All questions had four options each.

***2. Distribution and collection:*** The data collection process involved in-person interactions with study participants who were explained the purpose and process of the survey, and were assisted in filling out the Google Forms on-site. This approach ensured that study participants understood the survey and provided their responses in a controlled environment. The sample size was calculated based on a 95% confidence interval, a 5% margin of error, and a 50% population to be gender specific. A total of 383 responses were collected for the quantitative part, with 266 from female patients and 117 from male patients. For qualitative data, data saturation was observed after thirty interviews but we continued collection to ensure equal representation of both genders (25 male and 25 female). Data collection was conducted for six months to allow sufficient time for capturing a representative sample of responses. The research team was responsible for collecting and managing the data, ensuring all responses were accurately recorded.

### Data analysis:

***1. Quantitative analysis:*** Descriptive statistics were employed to summarize the demographic characteristics of study participants and their preferences. Frequency distributions and percentage analyses were conducted to identify patterns and trends in patient preferences related to dentist attributes and gender predispositions while the association between the patient’s preferences and dentist-associated and patient-associated characteristics was studied using the help of the Chi-square test. The significance level was kept at P ≤ 0.05. Statistical analysis was performed using SPSS software version 26.0 (IBM Corporation, SPSS Inc., Chicago, IL, USA).

***2. Qualitative analysis:*** The qualitative data was collected employing face-to-face sessions comprising semi-structured interview questions by a single researcher with the study participants. The interview questions guide was prepared by the researchers earlier and validated by two experts. Interviews were audio recorded by the record function of a mobile phone device (Samsung S22) and notes were also taken during the process. To accurately capture the essence of participants’ perceptions, they were encouraged to answer in Urdu (Pakistan’s national language), their mother tongue, or their first language. The data was securely recorded and then translated and transcribed verbatim.

Responses to open-ended questions were analyzed thematically by transcribing audio recordings of study participants to identify recurring themes and sentiments related to gender predispositions in dental treatment. After transcribing the data, codes were identified manually based on current issues that emerged from the data. These codes were organized into themes and sub-themes. The Braun and Clarke framework was used for thematic analysis and an inductive approach was used.^10^ To ensure the trustworthiness of our research, participants were also requested to verify the transcripts while to conceal the identities of participants in the audio recordings and transcription verbatim, they were given pseudonyms of ID- 1-25 for males and ID- 26-50 for females. Triangulation of data was also ensured by a thorough review of transcripts by two researchers.

## RESULTS

This study examined gender predispositions in patient preferences regarding dental treatment and a total of 383 participants were surveyed, comprising 69% females (n=266) and 31% (n=117) males to assess their attitudes towards various dentist attributes.

### Quantitative findings:

The analysis revealed that the gender of study participants and education level significantly influence patient perceptions and preferences in dental treatment (Table-I). In Table-II, distinct patterns in preferences related to dental professional characteristics. The gender of the dental professional impacts patients’ views and significant associations were found linked to attention to aesthetics (p ≤ 0.04) and concern for cleanliness (p ≤ 0.03) with female dentists perceived more favorably in these areas. Additionally, patients demonstrate clear gender preferences when it comes to multiple visits (p ≤ 0.00) suggesting that trust, comfort, and communication are strongly influenced by the dentist’s gender.

**Table-I:** Distribution of Age & Level of Education.

Age (Years)	Level of Education						
	Middle Level (%)	Matriculation (%)	Intermediate (%)	Bachelors (%)	Masters (%)	Uneducated (%)	Total (%)
0 - 12	01 (0.2%)	05 (1.3%)	05 (1.3%)	04 (1.0%)	02 (0.5%)	0 (0%)	17 (4.4%)
18 - 30	01 (0.2%)	10 (2.6%)	31 (8.1%)	192 (50.1%)	40 (10.4%)	02 (0.5%)	276 (72.1%)
36 - 48	03 (0.7%)	08 (2.0%)	10 (2.6%)	21 (5.4%)	20 (5.2%)	04 (1.0%)	66 (17.2%)
54 - 66	01 (0.2%)	04 (1.0%)	01 (0.2%)	04 (1.0%)	10 (2.6%)	03 (0.7%)	23 (6.0%)
72 - 84	0 (0%)	01 (0.2%)	0 (0%)	0 (0%)	0 (0%)	0 (0%)	01 (0.2%)
TOTAL	06 (1.5%)	28 (7.3%)	47 (12.2%)	221 (57.7%)	72 (18.7%)	09 (2.3%)	383

**Table-II:** Patient Perceptions & Preferences Regarding Dental Professional Characteristics.

Questions	Gender				
	Either Male or Female (%)	Female (%)	Male (%)	I Can’t Say (%)	P-Value
Appears more experienced	142 (37%)	32 (8%)	196 (51%)	13 (3%)	0.156
Has better time management skills	142 (37%)	73 (19%)	155 (40%)	13 (3%)	0.586
Concern for cleanliness	172 (44%)	130 (33%)	59 (15%)	22 (5%)	0.035*
Pays more attention to aesthetics	132 (34%)	167 (43%)	60 (15%)	24 (6%)	0.040*
Better at maintaining confidentiality	160 (41%)	64 (16%)	134 (34%)	25 (6%)	0.572
Spends more time suggesting preventive measures	143 (37%)	125 (32%)	89 (23%)	26 (6%)	0.061
Decisive and confident about the choice of treatment	185 (48%)	51 (13%)	141 (36%)	6 (1%)	0.169
Charges more fee for treatment offered	135 (35%)	46 (12%)	137 (35%)	65 (16%)	0.104
Whom do you prefer for multiple visits	160 (41%)	102 (26%)	113 (29%)	8 (2%)	0.000*
More organized and well-mannered	174 (45%)	117 (30%)	83 (21%)	9 (2%)	0.390

* P < 0.05 was considered a significant, Statistical analysis was performed by applying the Chi-Square Test.

Level of education also plays a critical role as patients with a higher level of education tend to feel more confident in their treatment choices. In Table-III patient-related factors, highlighting trends in preferences. This analysis identifies specific patterns in patient choices and priorities for each area of inquiry. These findings highlight how demographic factors shape patient expectations, emphasizing the importance of tailored communication and care strategies in dentistry to enhance patient satisfaction and trust. Significant associations were also found for patient-related factors regarding sharing dental fears (p ≤ 0.00), discussing general health issues (p ≤ 0.00), discussing financial issues (p ≤ 0.00), and understanding problems better (p ≤ 0.00).

**Table-III:** Patient Perceptions & Preferences Regarding Patient-Associated Factors.

QUESTIONS	GENDER				
	Either Male or Female (%)	Female (%)	Male (%)	I Can’t Say (%)	P-Value (%)
Better communication skills	185 (48%)	105 (27%)	87 (22%)	6 (1%)	0.148
Seems to be more sincere with the patients	198 (51%)	99 (25%)	68 (17%)	18 (4%)	0.073
More comfortable in sharing dental fear & doubts	166 (43%)	134 (34%)	80 (20%)	3 (0.7%)	0.000*
More comfortable about answering questions about general health	147 (38%)	150 (39%)	80 (20%)	6 (1%)	0.000*
More comfortable to discuss financial limits for treatment option	183 (47%)	107 (27%)	74 (19%)	19 (4%)	0.000*
Who listen & understands problem better	167 (43%)	124 (32%)	83 (21%)	9 (2%)	0.005*
Who is more sensitive & caring	114 (29%)	201 (52%)	61 (15%)	7 (1%)	0.825
Who seems to be less emotional & more practical	104 (27%)	49 (12%)	216 (56%)	14 (3%)	0.767

* P < 0.05 was considered a significant, Statistical analysis was performed by applying the Chi-Square Test.

### Qualitative findings:

The perceptions of the study participants can be described as presented in Table-IV. The thematic analysis addresses five prevalent themes and twelve sub-themes which include comfort level, perception of competence, cultural and religious factors, trust and confidence in treatment, bias and stereotypes.

**Table-IV:** Themes and Subthemes of Qualitative Analysis.

THEMES	SUB-THEMES
Theme 1: Comfort Level	Sub-theme 1.1: Male Patients’ Comfort with Male Dentists
	Sub-theme 1.2: Female Patients’ Comfort with Female Dentists
Theme 2: Perception of Competence	Sub-theme 2.1: Male Dentists Perceived as More Competent
	Sub-theme 2.2: Female Dentists Perceived as More Empathetic
	Sub-theme 2.3: Equal Competence Regardless of Gender
Theme 3: Cultural and Religious Factors	Sub-theme 3.1: Influence of Parda (Veil) on Gender Preference
	Sub-theme 3.2: Cultural Expectations of Same-Gender Practitioners
Theme 4: Trust and Confidence in Treatment	Sub-theme 4.1: Trust in Same-Gender Dentists
	Sub-theme 4.2: Perceived Empathy and Care from Female Dentists
Theme 5: Bias and Stereotypes	Sub-theme 5.1: Bias Against Female Dentists
	Sub-theme 5.2: Stereotypes of Male Dentists as More Competent
	Sub-theme 5.3: No Gender Bias (Equal Competence)

### Theme 1: Comfort level:

The comfort level of patients with dental professionals of the same gender was a recurring theme in the responses. Male patients often expressed a preference for male dental professionals, citing ease of communication and familiarity.

One participant noted, *“I feel more at ease discussing my concerns with a male dentist; it feels more natural.”* (ID-3)

This sentiment was mirrored by female patients, who generally felt more comfortable with female dental professionals, particularly for procedures involving prolonged physical interaction.

A female respondent shared, *“I prefer a female dentist because she understands my anxiety and makes me feel more at ease.”* (ID-27)

These responses indicate that gender plays a critical role in determining the comfort level of patients, which can impact their overall dental experience.

### Theme 2: Perception of competence:

The perception of competence varied significantly based on gender stereotypes. Many respondents viewed male dental professionals as technically more skilled, particularly in procedures requiring physical strength or precision.

One patient remarked, *“For complex procedures, I feel more confident with a male dentist because they seem more experienced.”* (ID-9)

Conversely, female dental professionals were often perceived as more empathetic and patient-centered.

A participant shared, *“My female dentist took the time to explain everything, which made me trust her more.”* (ID-29)

However, some patients expressed a neutral stance, emphasizing that competence should be judged by skills rather than gender.

One stated, *“I don’t think gender matters; what’s important is the dentist’s experience and professionalism.”* (ID-31)

This suggests that while gender partialities persist, there is a growing shift towards evaluating dental professionals based on ability.

### Theme 3: Cultural and religious factors:

Cultural and religious beliefs, particularly the practice of Parda (modesty), significantly influenced gender preferences in dentistry. Many female patients from conservative backgrounds preferred female dental professionals due to religious and cultural considerations.

One participant stated, “*I follow Parda, so I always prefer a female dentist.”* (ID-32)

Similarly, societal norms reinforced the idea that same-gender practitioners were more appropriate for treatment.

A respondent shared, *“My family insists that I go to a female dentist—it’s just how things are in our culture.”* (ID-47)

These findings highlight the deep-rooted influence of cultural and religious factors in shaping healthcare choices, which may limit patients’ access to a diverse range of professionals.

### Theme 4: Trust and confidence in treatment:

Trust in dentists was closely linked to gender, with many patients reporting higher confidence in same-gender practitioners. Male patients expressed trust in male dental professionals for handling surgical and technical procedures, whereas female patients felt female dental professionals were more considerate of their comfort.

One participant remarked, *“I trust a dentist of my gender more because they understand my concerns better.”* (ID-19)

Additionally, female dentists were frequently described as more empathetic and reassuring.

A respondent shared, *“She explained everything to me and made sure I was comfortable before starting.”* (ID-37)

These findings suggest that trust and confidence are shaped by personal experiences and gender-based perceptions, rather than actual differences in competence.

### Theme 5: Bias and stereotypes:

Despite the increasing presence of female dental professionals, gender predispositions persist in dentistry. Some patients hesitated to choose female practitioners for complex procedures, assuming male dentists were more skilled.

One respondent admitted, *“I was unsure about seeing a female dentist for a difficult procedure.”* (ID-21)

Conversely, female dental professionals were often expected to be more caring and communicative.

A patient remarked, *“I feel that female dentists are more understanding and take their time.”* (ID-38)

However, a subset of patients rejected gender-based predispositions altogether, focusing on experience and professionalism over gender.

One participant emphasized, *“A good dentist is a good dentist, regardless of gender.”* (ID-11)

These insights highlight the ongoing impact of stereotypes, while also indicating a gradual shift towards more equitable evaluations of dental professionals.

## DISCUSSION

Awareness of a patient’s gender predisposition can help a dental professional better fulfill or counter the patient’s expectations. The present study explored gender predispositions in dental treatment from the patient’s perspective. The study showed that most people generally did not prefer a specific gender when choosing their dentist. However, patients exhibited gender preferences when choosing their dentist for certain categories, believing that a specific gender would perform better in those situations, revealing their predispositions. A bilateral relationship between the patient and dental professional should ensure good-quality treatment, based on mutual respect, trust, and equity.^11^ Relevant works in the literature discuss similar concepts, where patients did not outright prefer a specific gender for dental treatment but categorized a dental professional based on gender norms influenced by various local psychosocial factors and personal experiences.^2^-^3^,^5^

In the present study, male dentists were considered more experienced, less emotional, and more practical in a professional environment as compared to female dentists, which aligns with findings in the literature.^2^ The reason for them being more experienced possibly could be due to gender norms, for instance, males are seen as breadwinners and have extended working hours as compared to female dentists, with their time and focus divided by household obligations, especially in South Asian cultures.^12^,^13^ The societal parameters of greater male representation in leadership roles, higher faculty positions, and greater encouragement to adopt confidence and assertive body language, which is often discouraged for women due to traditional social norms, may contribute to this predisposition.^14^,^15^ This tends to lead to long-standing stereotypes that view men as more competent and decisive in professional settings, which can influence patients’ confidence in treatment outcomes. Women are also more likely to be affected by imposter syndrome in workplace environments, therefore giving the conviction that male dentists are more experienced in comparison to their female colleagues.^16^

The present study further suggested that the study participants viewed male dentists as less emotional and more practical during treatment procedures, which is consistent with previous literature. This perception is perhaps influenced by cultural and social expectations that encourage men to be “generally” less emotional than women. This leads to the idea that men make decisions based on facts and logic, rather than personal sentiments. On the other hand, women are often seen as more emotionally expressive, among South Asians. However, Weigard et al. in their research suggest that men do not experience fewer emotions than women.^17^ They are often discouraged from showing vulnerability or emotional tendencies, as society expects them to fulfill the role of the hero and provider, focusing on problem-solving rather than emotional venting. Studies also indicate that men who displayed emotions were perceived as “weak” and their behavior is more critically evaluated in workplace environments as compared to women. Therefore, emotional expression in men may be context-dependent and influenced by various biopsychosocial factors, leading to the self-reported belief that men are less emotionally expressive.^18^

The current study additionally suggested that female dentists were considered more sensitive and caring which is in agreement with studies in the literature as compared to male dentists.^2^ Women are likely to show higher levels of empathy compared to men, but this is likely to be associated with motivation rather than an inherent biological system, due to acquired psychosocial pressures to be viewed as nurturing associated with traditional gender roles and power dynamics in interpersonal relationships. Previous studies show that empathy in individuals can be shaped by psychological and circumstantial influences connected to preconceived gender norms.^16^ Moreover, females are facially more expressive, often smiling more, which leads to greater trust and rapport with patients, giving the impression of being more sensitive and caring.^19^,^20^ These actions help reinforce positive emotions. However, self-perceived empathy and consideration may not necessarily reflect actual empathy levels in individuals, and may not be intrinsically linked to a specific gender.^15^

Patients also feel more comfortable answering questions about general health to a female dental professional.^3^,^5^ Since the majority of the data collected in the present study was from females, it was noticed that they felt more comfortable discussing their history with a female dental professional, due to a common perception that females spend more time conversing with patients. Both of these findings are consistent with studies that were come across in the literature.^3^,^5^ Furthermore, the present study analyzed whether cultural notions, shaped by personal experiences and education patterns, have changed in Pakistan over the past few years with more women entering the field of dentistry.^11^ A majority of the study participants remained impartial as most of the data was collected from qualified people and younger age groups, showing a shift in the traditional mindset of gender norms, expectations, and behaviors in Pakistan. However, gender predisposition regarding certain characteristics is still present as society places both men and women into frames based on conscious and subconscious realities and gender assumptions. Overall, gender predisposition has far-reaching implications for the healthcare sector and must be addressed both at the individual and societal levels. Patients and dental professionals should work together to create an environment that fosters respect, trust, and equal treatment for all, regardless of gender.

### Limitations:

The study is limited by its reliance on self-reported data, which may be subject to predilections or inaccuracies. The sample may also not be representative of the broader population due to the voluntary nature of participation and the specific hospital settings. The study also had certain limitations as data had more female participants as compared to males. Further research with a larger sample size needs to be done to further evaluate gender predispositions in the field of dentistry.

## CONCLUSION

This study showed that while gender predisposition influences certain aspects of dental treatment, the overall quality remains unaffected, with factors such as experience, cost, and treatment choice being prioritized. Addressing these predispositions is crucial for fostering equity and patient-focused care, ultimately leading to better outcomes and a more inclusive environment. Collaboration between patients and dental professionals is essential to ensure respect, trust, and equal treatment for all, regardless of gender. These findings also have significant implications for dental practice and policy.

By understanding patient preferences, dental professionals can tailor their approaches to improve satisfaction and care outcomes. Practices must actively address gender predispositions, ensuring equal recognition for both male and female dental professionals based on their skills and competencies. These insights can inform recruitment and training strategies within dental institutions, highlighting areas where gender predispositions are prevalent and focusing on developing preferred soft skills. Additionally, patients need to recognize these predispositions and actively work to counteract them. Patients should make a conscious effort to evaluate dental professionals based on their qualifications and abilities rather than their gender.

### Authors’ Contributions:

**AE, NA and AJK:** Contributed to the conceptualization of the study and is responsible for the integrity of the study.

**AE, EA and IA:** Helped in acquiring, analyzing, interpreting data, and writing the manuscript.

**NA, AJK and AS:** Contributed to the initial write-up in the introduction and discussion part.

**AE and AJK:** Revised it critically for important intellectual content.

All authors have read and approved the manuscript.

## References

[1] Hakami NA, Al-Musawa HI, Alharbi AI, Marwahi NA, Almutlaq AS, Alghamdi RA (2024). Public Preferences for Surgeon Gender in Saudi Arabia:A Cross-Sectional Analysis. Healthcare.

[2] Puranik MP, Kumar A (2015). Gender stereotypes in dental care:a cross-sectional study. Biomed Sci.

[3] Pandit N (2020). Patient-dentist relationship (the decisive factor for optimal results). J Indian Soc Periodontol.

[4] Alzahrani S, Aboalshamat K, Bedaiwi S, Alnefaie S, Almutairi T, Asiri S (2020). Patients'preferences for dentist's nationality and gender among residents of Jeddah, Saudi Arabia. Open Dent J.

[5] Prenetha R, Prabakar J (2022). A Cross-Sectional Hospital-Based Study On How Patients Perceive The Dental Care Provided By Male Or Female Dentists. J Adv Pharm Technol Res.

[6] Díaz-Narváez V, Oyarzún-Muñoz M, Reyes-Reyes A, Calzadilla-Núñez A, Martínez PT, González-Valenzuela C (2021). Psychometry and empathy levels and its dimensions in postgraduate students of dental specialties. Eur J Dent Edu.

[7] Friedlander AH (2004). Unique dental needs of gender-based populations. Spec Care Dentist.

[8] Pakistan population 2025.

[9] Raza A, Jauhar J, Abdul Rahim NF, Memon U, Matloob S (2023). Unveiling the obstacles encountered by women doctors in the Pakistani healthcare system:A qualitative investigation. PLoS One.

[10] Creswell JW, Clark VL (2017). Designing and conducting mixed methods research. SAGE.

[11] Govender V, Penn-Kekana L (2008). Gender biases and discrimination:a review of health care interpersonal interactions. Glob Public Health.

[12] Bussolo M, Ezebuihe JA, Muñoz Boudet AM, Poupakis S, Rahman T, Sarma N (2024). Social norms and gender disparities with a focus on female labor force participation in South Asia. World Bank Res Obs.

[13] Ayers KM, Thomson WM, Rich AM, Newton JT (2008). Gender differences in dentists'working practices and job satisfaction. J Dent.

[14] Eagly AH, Karau SJ (1991). Gender and the emergence of leaders:A meta-analysis. J Pers Soc Psychol.

[15] Kim A, Karra N, Song C, Linder PJ, Bonino F, Doig P (2024). Gender trends in dentistry:dental faculty and academic leadership. J Dent Educ.

[16] Price PC, Holcomb B, Payne MB (2024). Gender differences in impostor phenomenon:a meta-analytic review. Curr Res Behav Sci.

[17] Weigard A, Loviska AM, Beltz AM (2021). Little evidence for sex or ovarian hormone influences on affective variability. Sci Rep.

[18] Fischer A, LaFrance M (2015). What drives the smile and the tear:Why women are more emotionally expressive than men. Emot Rev.

[19] McDuff D, Kodra E, Kaliouby RE, LaFrance M (2017). A large-scale analysis of sex differences in facial expressions. PLoS One.

[20] LaFrance M, Hecht MA, Paluck EL (2003). The contingent smile:a meta-analysis of sex differences in smiling. Psychol Bull.

